# County-level CO_2_ emissions and sequestration in China during 1997–2017

**DOI:** 10.1038/s41597-020-00736-3

**Published:** 2020-11-12

**Authors:** Jiandong Chen, Ming Gao, Shulei Cheng, Wenxuan Hou, Malin Song, Xin Liu, Yu Liu, Yuli Shan

**Affiliations:** 1 School of Public Administration, Southwestern University of Finance and Economics, Chengdu, China; 2 School of Finance, Shanghai Lixin University of Accounting and Finance, Shanghai, China; 3 University of Edinburgh Business School, University of Edinburgh, 29 Buccleuch Place, Edinburgh, United Kingdom; 4 School of Statistics and Applied Mathematics, Anhui University of Finance and Economics, Bengbu, China; 5 Curtin University Sustainability Policy Institute, School of Design and the Built Environment, Curtin University, Perth, Australia; 6 nstitutes of Science and Development, Chinese Academy of Sciences, Beijing, 100190 China; 7 School of Public Policy and Management, University of Chinese Academy of Sciences, Beijing, 100049 China; 8 Integrated Research on Energy, Environment and Society (IREES), Energy and Sustainability Research Institute Groningen, University of Groningen, Groningen, 9747 AG Netherlands

**Keywords:** Environmental impact, Climate change, Carbon cycle

## Abstract

With the implementation of China’s top-down CO_2_ emissions reduction strategy, the regional differences should be considered. As the most basic governmental unit in China, counties could better capture the regional heterogeneity than provinces and prefecture-level city, and county-level CO_2_ emissions could be used for the development of strategic policies tailored to local conditions. However, most of the previous accounts of CO_2_ emissions in China have only focused on the national, provincial, or city levels, owing to limited methods and smaller-scale data. In this study, a particle swarm optimization-back propagation (PSO-BP) algorithm was employed to unify the scale of DMSP/OLS and NPP/VIIRS satellite imagery and estimate the CO_2_ emissions in 2,735 Chinese counties during 1997–2017. Moreover, as vegetation has a significant ability to sequester and reduce CO_2_ emissions, we calculated the county-level carbon sequestration value of terrestrial vegetation. The results presented here can contribute to existing data gaps and enable the development of strategies to reduce CO_2_ emissions in China.

## Abstract (summary)

**Table:** 

Measurement(s)	carbon dioxide emission • carbon dioxide sequestration
Technology Type(s)	machine learning
Factor Type(s)	temporal interval • geographic location
Sample Characteristic - Environment	carbon dioxide
Sample Characteristic - Location	China

Machine-accessible metadata file describing the reported data: 10.6084/m9.figshare.13090370

## Background & Summary

As one of the largest carbon emitters globally, China has pledged to reach the peak of its carbon emissions by 2030^1–3^, and significant effort has been put into developing a sustainable economy^4–6^. An increasing number of studies have focused on topics such as CO_2_ emissions accounts^7–9^, driving forces of CO_2_ emissions^10,11^, forecasting future emissions, and more^12,13^. However, most of this research has been conducted at the national, provincial^14–16^, or city level^17,18^. Actually, even within the same province or the same prefecture-level city, there can be obvious differences in CO_2_ emissions among counties. Research at the county-level is important for capturing regional heterogeneity and developing policies that can effectively lead to reductions in CO_2_ emissions.

Therefore, records of county-level CO_2_ emissions are required, which could help to fill the gaps in China’s CO_2_ emission data and could be used for the development of strategic policies that propose county specific emission reduction actions. However, very few studies have investigated the county-level emissions in China owing to low availability of data sources, and the corresponding studies have limitations in methodology, time span, and geographical coverage. These studies can be classified into two main categories:

1. Previous studies calculated the county-level CO_2_ emissions on the basis of the published energy use data^19,20^. For example, Cai *et al*.^19^ estimated CO_2_ emissions of 16 counties in Tianjin, China in 2007 based on the construction of a CO_2_ emissions grid, which was derived from the spatial distributions of energy use from the industrial sector, agricultural sector, and residential sector. Additionally, Guan *et al*.^20^ adopted the CO_2_ emission coefficients provided by the IPCC and 11 types of energy use, such as coal, coke, coal gas, and natural gas, to calculate the CO_2_ emissions of 18 counties in the Ningxia Hui Autonomous Region during 1991–2011. The county-level CO_2_ emissions estimated by these studies relied on the published energy use data. Hence, the estimated emissions are always limited to a small coverage and short period, owing to the insufficient and often incomparable county-level energy use information.
2. When dealing with the lack of data in cities and counties, some studies used a top-down approach to calculate their CO_2_ emissions^21,22^. Although some scholars have used several socioeconomic variables (such as urbanization ratio and population density) as the cutting index^23^; also the nighttime light data are always selected as proxies to downscale the total energy-related CO_2_ emissions^21,22^. For example, Meng *et al*.^21^ used the brightness data from Defense Meteorological Satellite Program/Operational Linescan System (DMSP/OLS) satellite imagery to downscale and estimate CO_2_ emissions of 287 prefecture-level cities in China during 1995–2010. Similarly, Su *et al*.^22^ calculated city-level CO_2_ emissions in China during 1992–2012 based on the DMSP/OLS imagery. In the present case, there are primarily two reasons for selecting lighting data: first, the brightness distribution on the surface of the earth at night is closely related to human activities; second, with advancements in remote sensing interpretation technologies, a wider time-span and coverage of global lighting data can be extracted and used. In addition, considering the advantages above, nighttime light data have also been widely accepted and applied in other research fields, such as energy use forecasting, gross domestic productivity evaluation, and population distribution estimation^24–26^.

Thus, based on the available nighttime light data provided by DMSP/OLS images, China’s energy-related CO_2_ emissions can be calculated at micro-level administration. However, because the DMSP/OLS images are only available up to 2013, the research period was limited. Additionally, even though Suomi National Polar-Orbiting Partnership/Visible Infrared Imaging Radiometer Suite (NPP/VIIRS) images provide another source of nighttime light brightness data after 2012, the evident gaps between the two sets of satellites’ data have hindered the construction of long-term nighttime light data sets and calculations of CO_2_ emissions. Thus, several studies have made attempts to unify the two sets of satellite data^27–29^. But matching the results proved difficult, and other problems involving discontinuity and saturation were encountered. Hence, there is room for further improvements.

In addition, existing literature on CO_2_ emissions reduction has only focused on the energy-related carbon emissions, and the influence of carbon sequestration of vegetation have always been ignored. With regard to the concept of plant carbon sequestration capacity, it is a natural carbon sequestration process, which directly counteracts the processes of emitting CO_2_ into the atmosphere. In addition, the natural processes mainly originate from vegetation net primary productivity (NPP) or net ecosystem productivity– vegetation in the ecosystem absorb CO_2_ from the air, produce carbohydrates such as glucose through photosynthesis, and release oxygen. Actually, vegetation has a significant effect on CO_2_ sequestration, and can account for a major part of the CO_2_ emissions associated with energy use. Among them, terrestrial vegetation plays a significant role in CO_2_ sequestration, and methods of estimation of its sequestration capacity have advanced and have been widely adopted^29^. Therefore, we also estimate the county-level carbon sequestration values of terrestrial vegetation, which facilitates more comprehensive research on reducing CO_2_ emissions in China and evaluating sustainable develoment^30^.

Thus, our present study makes the following marginal contributions to this field of research: (1) we developed a new model and employed a particle swarm optimization-back propagation (PSO-BP) algorithm to unify the scale of DMSP/OLS and NPP/VIIRS images during 1997–2017, which obtain superior fitting effects than those of previous studies based on original models and normal econometrics; (2) we adopted the PSO-BP algorithm to downscale the provincial energy-carbon emissions based on the nighttime light data, and calculated 2,735 county-level energy-related carbon emissions during 1997–2017; and (3) we estimated the corresponding county-level carbon sequestration values of terrestrial vegetation, which is an issue that has rarely been considered in previous studies and plays a significant role in CO_2_ emissions mitigation.

## Methods

### Study areas and data preprocessing

Since China is one of the largest CO_2_ emitter globally, our aim was to facilitate the determination of carbon reduction status in China and to address current data gaps in China. In addition, our research results could facilitate energy saving and emissions reduction activities and efforts in other countries, especially in other developing countries.

As the most basic governmental unit in China, counties should play important roles in the implementation of emission reduction policies from the central, provincial, and municipal governments. Therefore, we selected county-level CO_2_ emissions as a research focus. Our study areas cover 2,735 counties of 30 provinces in China mainland (excluding Tibet, Hong Kong, Macau, and Taiwan) based on the accessibility of data sets, which cover approximately 87% of China’s land area, over 90% of the population, and 90% of the GDP.

Our estimated and provided data sets include energy-related CO_2_ emissions (1997–2017) and carbon sequestration values for terrestrial vegetation (2000–2017). The estimated boundary for energy-related CO_2_ emissions lies in Scope 2 proposed by the Climate Action Registry (CAR) and other institutes, which is consistent with Meng *et al*.^21^, Su *et al*.^22^, and Zhao *et al*.^28^.

Three satellite data sets were used in our study: two types of nighttime light data (provided by DMSP/OLS^31,32^ and NPP/VIIRS^33^ images) and net primary productivity data of terrestrial vegetation (which were provided by the MODIS NPP products^34^). Considering that there are several problems in the satellite images, such as discontinuities, the white noise, and fill values, these data sets need to be pre-processed before they can be further used.

The DMSP/OLS images were from the period of 1992–2013, and these data had problems stemming from discontinuities, saturation, and incomparability. Hence, we adopted several methods including inter-calibration, radiometric calibration, intra-annual composition, and inter-annual series correction methods proposed by previous studies to obtain continuous and stable DMSP/OLS images^27,28,35^.

We used the monthly NPP/VIIRS images, an approach consistent with previous studies^26,27^. Additionally, because of the influence of stray light pollution, lighting data in the mid–high latitudes of China in summer showed large errors; thus, we removed the images from June to August, and we used the remaining monthly data to synthesize the annual data. Then, we applied a Gaussian low-pass filter with a window size of 5 × 5 to mitigate the NPP/VIIRS images’ spatial variability and smooth the data to better match the DMSP/OLS images^36^. The *σ* was set as 1.75 in accordance with studies by Li *et al*.^37^ and Zheng *et al*.^38^ Moreover, to further reduce the white noise of NPP/VIIRS images, we replaced the negative values with zero and set a threshold of 0.3 nW·m^−2^·sr^−1^ in the annual images, which is consistent with earlier work^36^.

The MOD17A3 products provided by the National Aeronautics and Space Administration (NASA) have fill values and need to be multiplied by a 0.0001 conversion factor. Next, following the user guides^39,40^, we obtained the net primary productivity data. Finally, based on the conversion coefficient (i.e., 1.62/0.45) used by Chen *et al*.^30^, we obtained the carbon sequestration values of terrestrial vegetation, including Evergreen Needleleaf Forest (ENF), Evergreen Broadleaf Forest (EBF), Deciduous Needleleaf Forest (DNF), Deciduous Broadleaf Forest (DBF), Mixed forests (MF), Closed Shrublands (CShrub), Open Shrublands (OShrub), Woody Savannas (WSavanna), Savannas (Savanna), grassland (Grass), and Croplands (Crop). Based on vector cutting, we obtained the county-level nighttime light values and carbon sequestration values of vegetation. The vector map of county-level cities in China in 2015 was derived from the National Geomatics Center of China, which is consistent with the work of Lv *et al*.^27^ and Zhao *et al*.^28^.

### Inter-calibration between DMSP/LOS and NPP/VIIRS Based on PSO-BP

Because the DMSP/OLS and NPP/VIIRS images were derived from different types of satellites, there are evident gaps in the two sets of data. Specifically, there are discrepancies caused by various factors such as the use of different sensors, different spatial resolutions, different spread functions, and so on^36^. However, the mechanisms for explaining the differences remain like a “Black Box,” and any fixed functional form for the inter-calibration between DMSP/OLS and NPP/VIIRS data may fail to produce a good match between the two sets of data and lead to large errors. Therefore, in the present study, we used an artificial neural network (ANN) to explore the relationship between the DMSP/OLS and NPP/VIIRS data rather than conventional econometric methods, because the conventional methods often fail to model the non-linear relationships^41^.

Additionally, because the back-propagation (BP) algorithm has performed well in previous studies for constructing regressions and obtaining local optimistic results^42,43^, the BP algorithm was adopted in this research. However, given that the BP algorithm can lead to data at local extremes and training failures, we also combined the BP algorithm with particle swarm optimization (PSO)—PSO has shown great potential in exploring the global optimistic results^44,45^.

As for the input parameters, we followed the approach of Zhao *et al*.^28^ and selected the county-level mean pixel values of NPP/VIIRS in 2013 ($V$) as the input. Given the geographical heterogeneity of individual data in mainland China, we made use of the minimum boundary method to obtain each county’s central geographic coordinates and used Arcmap 10.5 to obtain the area of each county. Then, we selected the central geographic coordinates ($X$ and $Y$) and the area of each county ($A$) as the supplementary input parameters, which greatly improved the matching accuracy of the two sets of data and reduced errors. To enhance the accuracy of modelling, we use the logarithmic form of the input parameters according to the suggestion of Li *et al*.^37^ In addition, with regard to the output parameter, we select the county-level mean pixel values of DMSP/OLS in 2013 (*D*).

Based on initializing the ANN weights with the PSO technique, we set values of *C*_1_ and *C*_2_ both as 2.0, the maximum iteration number as 50, and the population size as 20^45,46^. Additionally, the structure of the model was set as one hidden layer with five nodes in the hidden layer, which is consistent with the work of Mohamad *et al*.^45^ The total number of samples was 2,826. Among these, 2,000 samples were randomly selected as training samples, whereas the other 826 samples were used as testing samples. The calculation procedure for the PSO-BP model was consistent with the work of Mohamad *et al*.^45^ and Yin *et al*.^47^.

**Fig. 1:**
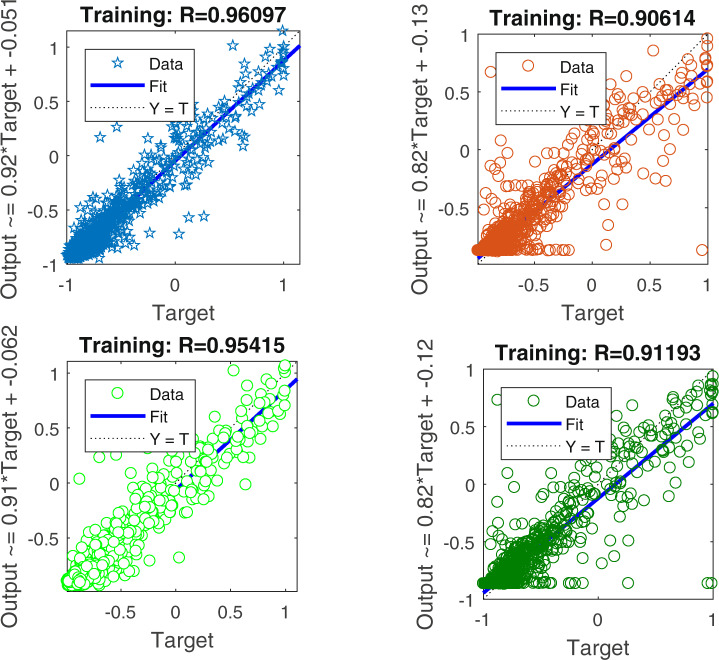
Training results for the mean pixel values in 2013. Data represent (**a**) the results with the supplementary input parameters based on the particle swarm optimization-back propagation (PSO-BP) algorithm, (**b**) the results for only the mean pixel values of nighttime light values based on the PSO-BP algorithm, (**c**) the results with the supplementary input parameters based on the BP algorithm, and (**d**) the results with only the mean pixel values of nighttime light values based on the BP algorithm.

To test the validity of the PSO-BP algorithm and the proposed supplementary input parameters, we also trained the BP algorithm and corresponding algorithm without the supplementary input parameters as control groups. The best training results are presented in Fig. 1. As shown in Fig. 1, all of the correlation coefficient values for the training results of mean pixel values in 2013 were more than 0.9, which indicated that the ANN was advantageous for identifying the potential relationship between DMSP/OLS and NPP/VIIRS data. Among the results, it was evident that the models that considered geographic coordinates and area (i.e., panels a and c) showed comparably better fitting effects than models that only used the county-level mean pixel values of NPP/VIIRS data as input parameters (i.e., panels b and d). Additionally, the correlation coefficient values for the PSO-BP algorithm were higher than those for the BP algorithm, thus indicating that the PSO-BP algorithm was better for determining the potential matching relationship between DMSP/OLS and NPP/VIIRS images.

**Fig. 2:**
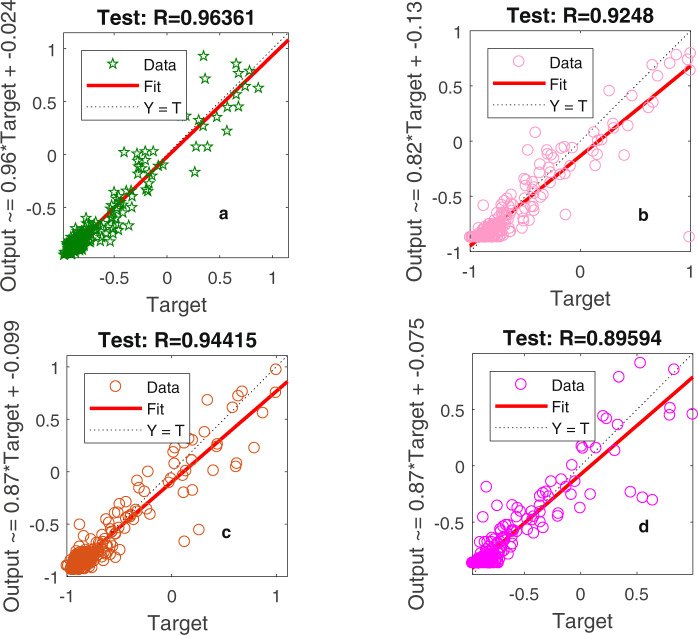
Test results for the mean pixel values in 2013. Data represent (**a**) the results with the supplementary input parameters based on the particle swarm optimization-back propagation (PSO-BP) algorithm, (**b**) the results with only the mean pixel values of nighttime light values based on the PSO-BP algorithm, (**c**) the results with the supplementary input parameters based on the BP algorithm, and (**d**) the results with only the mean pixel values of nighttime light values based on the BP algorithm.

Figure 2 shows the test performances of the four models, and these results can be used to identify the fitting effects of each model. The highest correlation coefficient value of 0.96361 in model *a* for the testing dataset indicated that the proposed PSO-BP algorithm reliably matched the DMSP/OLS images with NPP/VIIRS images in later years (e.g., 2014 and 2015). The correlation coefficient, R^2^, based on our method, was 0.955, which was significantly higher than values obtained previously, including the 0.8354 of Lv *et al*.^27^, 0.9154 of Zhao *et al*.^28^, and 0.91 of Li *et al*.^36^.

Furthermore, the matching work has not yet been completed. Although the correlation coefficient is close to 1, there were evident and unavoidable faults in some counties in 2013 (i.e., DMSP/OLS data in 2013) and 2014 (i.e., converted NPP/VIIRS data in 2014), which also exist in previous studies. Therefore, we make use of the annual increase amounts of converted NPP/VIIRS data during 2013–2017 to obtain the final simulated DMSP/OLS data during 2014–2017, avoiding the shortcoming of faults and discontinuities in some regions during 2013–2014.

In summary, based on the PSO-BP algorithm, we could confidently convert the scale of NPP/VIIRS data during 2013–2017 to the scales of DMSP/OLS data and obtain stable and continuous county-level nighttime light data during 1997–2017, which lay a foundation for further calculations of county-level CO_2_ emissions.

### Calculation of CO_2_ emissions based on satellite data

Because provincial energy balance tables were available and there was a lack of energy use data for various counties, we first established the relationship between provincial CO_2_ emissions and nighttime light data (i.e., the sum of the DN values) in this study; then, the sum of the DN values was used as a proxy to estimate the county-level carbon emissions.

First, the estimations of provincial CO_2_ emissions were carried out based on the following method provided by the Intergovernmental Panel on Climate Change (IPCC), which has been widely adopted^4,48,49^:

1 $${C}_{E}^{t}=\mathop{\sum }\limits_{i=1}^{30}{C}_{Direct,i}^{t}=\mathop{\sum }\limits_{i=1}^{30}\mathop{\sum }\limits_{j=1}^{17}\left[{E}_{ij}^{t}\times LC{V}_{ij}^{t}\times C{C}_{ij}^{t}\times CO{F}_{ij}^{t}\times \frac{44}{12}\right]$$

where ${C}_{E,i}^{t}$ represents the provincial CO_2_ emissions from energy use (unit: million tons); ${E}_{ij}^{t}$ represents the *j*^*th*^ type of energy use in province *i*; $LC{V}_{ij}^{t}$ is the low calorific value of the *j*^*th*^ energy consumption; $C{C}_{ij}^{t}$ is the carbon content of the *j*^*th*^ energy source; and $CO{F}_{ij}^{t}$ is the carbon oxidation factor of the *j*^*th*^ energy source. In addition, 17 types of fossil fuel used are considered, including raw coal, cleaned coal, other washed coal, briquettes, gangue, coke, coke oven gas, blast furnace gas, converter gas, other gases, other coking products, crude oil, gasoline, kerosene, diesel oil, fuel oil, naphtha, lubricants, paraffin, white spirit, bitumen asphalt, petroleum coke, other petroleum products, liquefied petroleum gas (LPG), refinery gas, and natural gas.

Subsequently, to avoid spurious regression problems, we adopted the unit root test to verify the relationship between provincial CO_2_ emissions *c*, and sum of DN values *sdn*. The results are presented in Table 1. It was evident that the sum of DN values and carbon emissions had to be processed at the same time with Eq. (1). Then, the co-integration Pedroni test was adopted^50^, which has been widely accepted in the field of econometrics^51–53^. The majority of tests led to the rejection of the null hypothesis of no co-integration, thus suggesting that there was significant co-integration among the provincial carbon emissions and sum of DN values.

**Table 1:** Results for the panel unit root tests and co-integration tests.

Variables	LLC		IPS	
	Level	First difference	Level	First difference
sdn	0.7541	−8.0742***	12.3274	−7.2143***
	(0.7746)	(0.0000)	(1.0000)	(0.0000)
c	−2.0716**	−5.2931***	7.0507	−8.2347 ***
	(0.0191)	(0.0000)	(1.0000)	(0.0000)
**Pedroni test**	**Panel PP statistic**	**Panel ADF statistic**	**Group PP statistic**	**Group ADF statistic**
	2.4199***	5.2237***	1.2318	3.7719***
	(0.0078)	(0.0000)	(0.1090)	(0.0001)

Notes: (1) Values in parentheses are the p-values; LLC denotes the Levin, Lin, and Chu t test, while IPS denotes the Im, Pesaran, and Shin Wald statistic test. (2) The LLC and IPS tests for all series include an intercept term. (3) The ** and *** symbols denote rejection of the null hypotheses of a unit root at the 5% and 1% significance level, respectively.

**Fig. 3:**
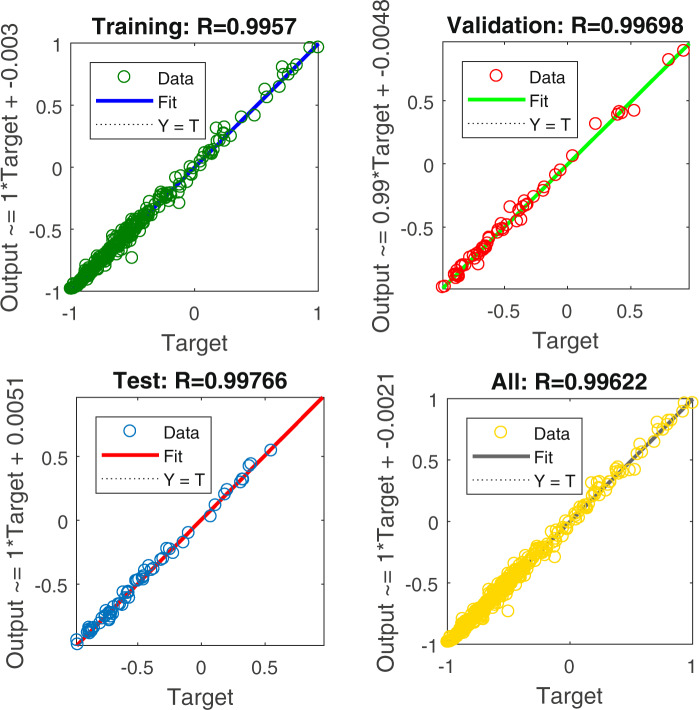
Training and test results for the relationship between provincial carbon emissions and the sum of digital number (DN) values.

Furthermore, considering that the relationship between provincial CO_2_ emissions and nighttime light data is non-linear, the normal econometric methods may lead to relatively high errors^27^; here, we employed the PSO-BP algorithm to fit and train the relationship. We selected the sum of DN values, dummy variables of identity, and year as the input parameters, and the provincial CO_2_ emissions were the output parameter. In addition, the other initialized parameters were consistent with those discussed in the earlier section on the inter-calibration. The results are presented in Fig. 3.

The test and training results showed great fitting effects, which were indicative of the high effectiveness of the algorithm. Notably, the coefficient of determination R^2^ of 0.9895 was higher than the 0.94 of Meng *et al*.^21^ and 0.6922 of Lv *et al*.^27^ estimated based on conventional econometric methods. Then, based on the concept of the top-down method and a DN value-based weighted-average strategy^21,22,41^, we obtained the county-level carbon emissions.

## Data Records

A total of 5470 data records (county-level CO_2_ emissions caused by energy use and carbon sequestration values of terrestrial vegetation). Among them, there were 2,735 CO_2_ emission county data records associated with energy use (1997–2017), and corresponding 2,735 carbon sequestration values associated with terrestrial vegetation (2000–2017).

**Fig. 4:**
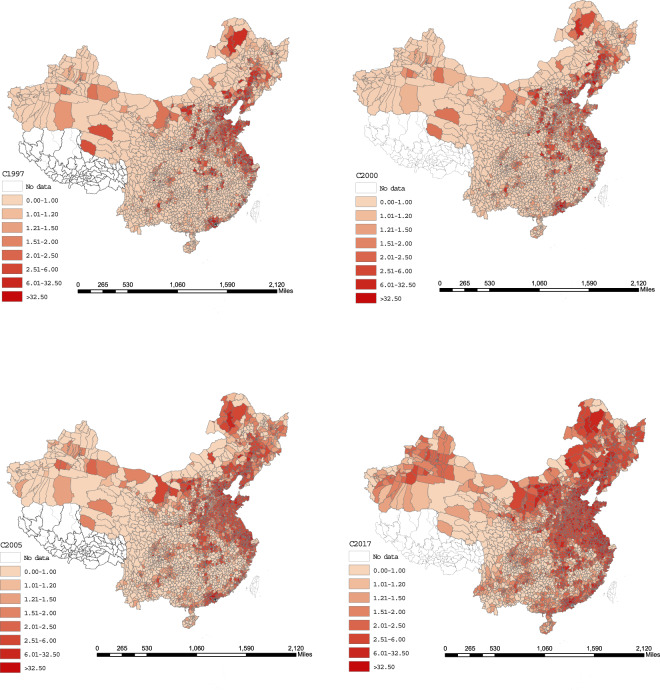
China mainland’s county-level CO_2_ emissions from energy combustion in 1997, 2000, 2005 and 2017 (unit: million tons).

**Fig. 5:**
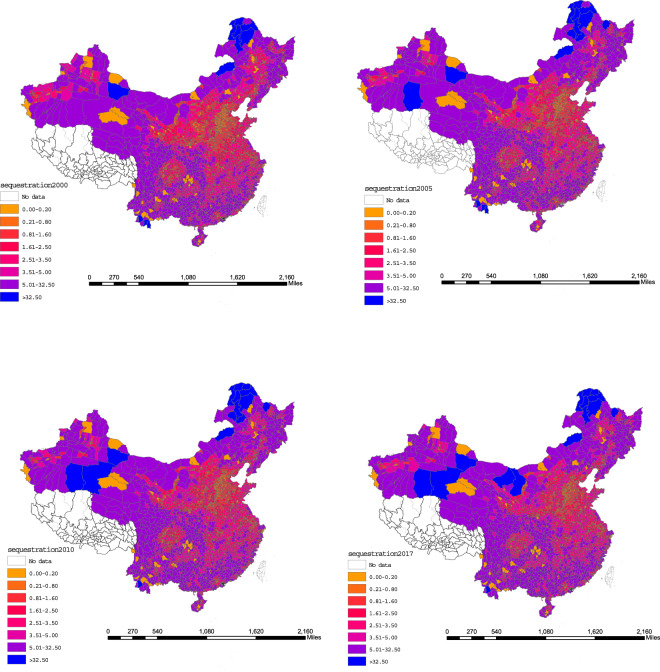
China mainland’s county level spatial and temporal patterns of carbon sequestration capacity of terrestrial vegetation in 2000, 2005, 2010, and 2017 (unit: million tons).

The present dataset and vector map are made public under Figshare^54^. And the units are million tons. The temporal and spatial changes in the China mainland’s county-level energy-related CO_2_ emissions and the carbon sequestration value of terrestrial vegetation are presented in Figs. 4 and 5.

## Technical Validation

### Validity testing for temporal and spatial nighttime light data changes

**Fig. 6:**
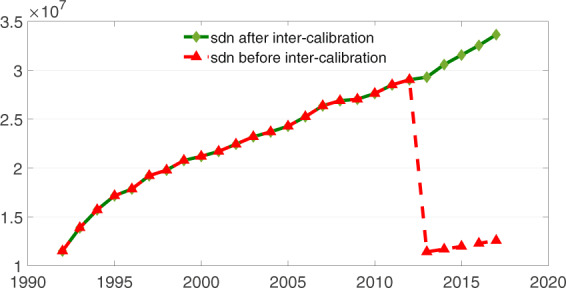
Total trend for the sum of DN values (*sdn*) during 1992–2017 (unit of the sum of DN values: 10^7^).

On the basis of the inter-calibration method described earlier, we were able to obtain continuous and stable county-level nighttime light DN values during 1997 to 2017, and the sum of DN values is presented in Fig. 6. The red line represents the changes in the sum of DN values before the inter-calibration between DMSP/OLS and NPP/VIIRS images, and the green line represents the changes in the sum of DN values (*sdn*) after the inter-calibration based on the PSO-BP algorithm. Evidently, there was a gap between the scale of DMSP/OLS and NPP/VIIRS images before the matching. Additionally, the trend of our inter-calibrated results continuously increased, which is consistent with previous studies^27,28^.

Subsequently, because nighttime light data tend to be highly consistent with economic output^55,56^ and power consumption data^57–59^, we used the provincial cross-sectional gross domestic product (GDP) and power consumption to individually perform linear regressions with the sum of DN values (*sdn*) during 1997–2017. These results are presented in Table 2.

**Table 2:** Validity test results for the spatial patterns of nighttime light data based on GDP and power consumption.

Year	Model (1)			Model (2)		
	Slope	AIC values	R^2^	Slope	AIC values	R^2^
1997	0.0036 (0.0000)	17.03	0.87	0.0005 (0.0000)	12.62	0.92
1998	0.0038 (0.0000)	17.13	0.87	0.0005 (0.0000)	12.74	0.91
1999	0.0039 (0.0000)	17.23	0.88	0.0005 (0.0000)	12.86	0.91
2000	0.0043 (0.0000)	17.45	0.88	0.0006 (0.0000)	12.96	0.92
2001	0.0047 (0.0000)	17.65	0.88	0.0006 (0.0000)	13.08	0.92
2002	0.0051 (0.0000)	17.83	0.88	0.0007 (0.0000)	13.30	0.93
2003	0.0058 (0.0000)	18.08	0.89	0.0008 (0.0000)	13.70	0.92
2004	0.0068 (0.0000)	18.41	0.89	0.0009 (0.0000)	13.97	0.92
2005	0.0080 (0.0000)	18.75	0.90	0.0010 (0.0000)	14.20	0.92
2006	0.0091 (0.0000)	19.04	0.90	0.0011 (0.0000)	14.42	0.93
2007	0.0104 (0.0000)	19.37	0.90	0.0012 (0.0000)	14.61	0.93
2008	0.0122 (0.0000)	19.65	0.91	0.0012 (0.0000)	14.70	0.93
2009	0.0132 (0.0000)	19.90	0.90	0.0013 (0.0000)	14.85	0.93
2010	0.0154 (0.0000)	20.23	0.90	0.0015 (0.0000)	15.15	0.93
2011	0.0177 (0.0000)	20.53	0.90	0.0016 (0.0000)	15.34	0.93
2012	0.0191 (0.0000)	20.75	0.90	0.0016 (0.0000)	15.41	0.93
2013	0.0195 (0.0000)	21.11	0.88	0.0016 (0.0000)	15.87	0.91
2014	0.0201 (0.0000)	21.26	0.88	0.0016 (0.0000)	15.90	0.91
2015	0.0205 (0.0000)	21.42	0.88	0.0016 (0.0000)	15.93	0.92
2016	0.0214 (0.0000)	21.66	0.87	0.0016 (0.0000)	16.04	0.91
2017	0.0239 (0.0000)	21.94	0.87	0.0017 (0.0000)	16.16	0.91

Notes: The values in parentheses are p-values. Model (1) represents the linear regression of the provincial cross-sectional GDP with the sum of DN values; Model (2) represents the linear regression of the provincial cross-sectional power consumption with the sum of DN values (*sdn*). AIC denotes the Akaike information criterion.

With regard to the Model (1) results shown in Table 2, it was evident that there was a significant positive relationship between the provincial cross-sectional GDP and *sdn* during 1997–2017. All of the R^2^ values were over 0.87, and the AIC values were small, thus implying that inter-calibrated nighttime light data characterized the economic output well. Simultaneously, in regard to the Model (2) results shown in Table 2, the sum of the DN values were evidently consistent with the power consumption data because the slopes were significantly positive, the R^2^ values were high, and the AIC values were small.

### Validity testing for CO_2_ emissions based on satellite data

**Fig. 7:**
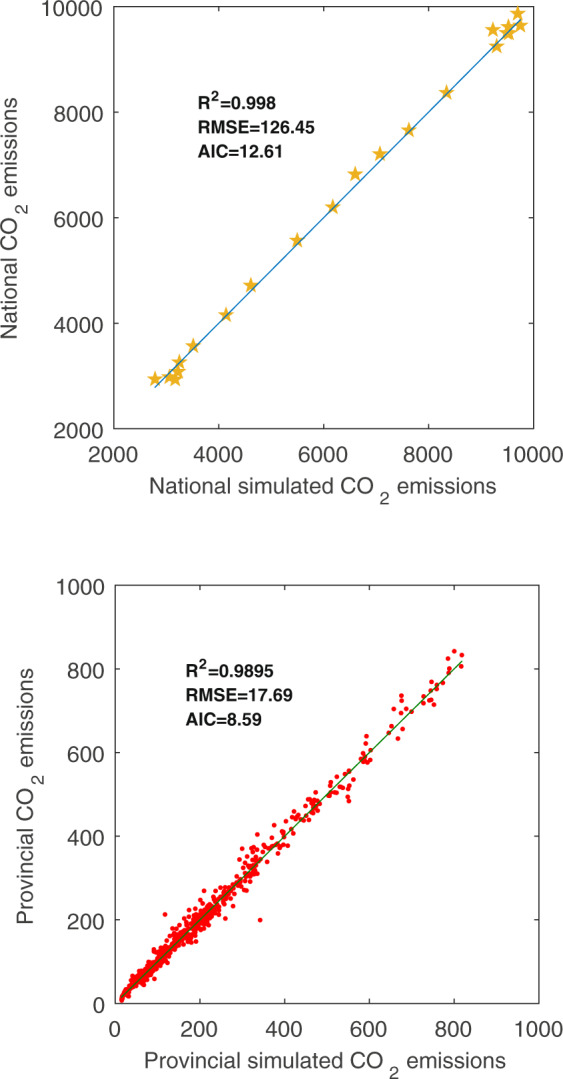
Scatter plots of our simulated national and provincial energy-related CO_2_ emissions with the CO_2_ emissions based on existing literature during 1997–2017 (unit: million tons).

The method of calculation of carbon sequestration value of terrestrial vegetation is consistent with that in Chen *et al*.^30^ and, therefore, was deemed a reliable measure. With regard to the validity of energy-related carbon emissions based on the nighttime light data, we made use of the national and provincial energy-related CO_2_ emissions provided by existing studies^17,60,61^ to conduct a comparison with the summary of our simulated energy-related carbon emissions. These results are presented in Fig. 7. Panels (a) and (b) in Fig. 7 individually show the scatter plots of our simulated national and provincial energy-related CO_2_ emissions with the CO_2_ emissions based on existing literature during 1997–2017. The results in each graph were highly consistent, thus indicating that the simulated CO_2_ emissions based on the nightlight data are reliable.

### Limitations and future work

Our datasets have several limitations, which we will address in the future to improve the accuracy of China’s county-level emission accounts. First, our estimated county-level CO_2_ emissions are based only on the nighttime light data, overlooking other factors such as urbanization rate, population, and earth surface temperature. Secondly, our estimated carbon sequestration values only include carbon sequestration capacity of terrestrial vegetation, without taking into account ocean carbon sequestration capacity^62^.

Therefore, our future work will include two aspects: first, we will combine night light data with other satellite data such as earth surface temperature provided by MOD11A2, and impervious surface data provided by Gong *et al*.^63^, to improve the accuracy of the calculated county-level carbon emissions. We will further analyze the carbon sequestration capacity of mangrove vegetation to enrich the estimated county-level carbon sequestration data.

## Usage Notes

First, the China mainland’s 2,735 county-level energy-related carbon emissions data is provided and has the advantages of wide coverage and long time-span. The data set can help fill the existing data gaps and be further used in future research. For example, scholars can use the data to further analyze the driving forces of the CO_2_ emissions at county-level rather than the nation^3^, province^4,5^ or prefecture-level^21,22,27^_._ Additionally, it can be used to further evaluate the emission reductions^19,20^ or construct budget allocation of CO_2_ emission rights in the county.

Second, considering that vegetation plays a significant role in sequestrating and reducing CO_2_ emissions, the dataset of the 2,735 county-level carbon sequestration values of vegetation can be combined with our provided county-level energy-related CO_2_ emissions. Evidently, the dataset could facilitate further comprehensive analyses and research on China’s emissions mitigation^30,64,65^, the gap between CO_2_ emissions and carbon sequestration, and comprehensive evaluation of sustainable development^65^.

Additionally, the present study was limited by differences in time spans of the energy-related CO_2_ emissions and carbon sequestration values for vegetation. Because of the availability of the original data, the energy-related CO_2_ emissions data span 1997–2017, while the carbon sequestration values of vegetation data span 2000–2017. Similarly, we have to point out that our study areas only include China mainland. The units of county-level CO_2_ emissions and carbon sequestration values provided are million tons.

## Supplementary information

Supplementary File 1

## Data Availability

The programs used to generate all the results were MATLAB (R2017b) and ArcGIS (10.5). The PSO-BP codes for matching the scales of the nighttime light data and modelling the relationships among the provincial energy-related CO_2_ emissions are presented in Suppl. File 1.
