# Supplementary material for: County-level CO2 emissions and sequestration in China during 1997–2017
Source: Sci Data. 2020 Nov 12;7:391. doi: 10.1038/s41597-020-00736-3 (PMC7665019; doi:10.1038/s41597-020-00736-3)
Supplement: Supplementary file 1 [file 41597_2020_736_MOESM1_ESM.doc]

1. **The PSO-BP codes for matching the scales of county-level mean pixel values from DMSP/OLS and NPP/VIIRS imagegs, which can be run by Matlab (R2017b).**

random_num = rand(1,2826);

[value,index] = sort(random_num);

x_train = input(index(1:2000),:)';

y_train = output(index(1:2000));

x_test = input(index(2001:2826),:)';

y_test = output(index(2001:2826));

%Set the number of BPNN nodes

inputnum = 4;

hiddennum = 5;

outputnum = 1;

%Set PSO related parameters

sizepop = 20;% population size

k = 50;% number of iterations

c1 = 2; c2 = 2;% learning factor

w = 0.8;% inertia factor

% Data normalization

[input_train,inputps] = mapminmax(x_train);

[output_train,outputps] = mapminmax(y_train);

% Determine the number of optimized parameters

length = inputnum*hiddennum+hiddennum+hiddennum*outputnum+outputnum;

param = rand(sizepop,length);

speed = rand(sizepop,length);

% Build BPNN

net = newff(input_train,output_train,hiddennum);

% Initialization (individual best position lbest | global best position gbest | individual fitness fitness | group best fitness fitnessbest)

for i=1:sizepop

fitness(i,:) = func(param(1,:),inputnum,hiddennum,outputnum,net,input_train,output_train);

lbest(i,:) = param(i,:);

end

[value,index] = min(fitness);

gbest = param(index,:);% group extreme position (parameter)

fitnessbest = value;% group extreme fitness (minimum MSE)

% Parameter optimization

for T=1:k

for i=1:sizepop

speed(i,:) = w*speed(i,:)+c1*rand*(lbest(i,:)-param(i,:))+c2*rand*(gbest-param(i,:)) ;

param(i,:) = param(i,:)+speed(i,:);

fit = func(param(i,:),inputnum,hiddennum,outputnum,net,input_train,output_train);

if fit<fitness(i,:)

fitness(i,:) = fit;

lbest(i,:) = param(i,:);

end

if fit<fitnessbest

gbest = param(i,:);

fitnessbest = fit;

end

end

MSE(T,:) = fitnessbest;

end

% Build a model and predict (gbest is the best parameter)

w1 = gbest(1:inputnum*hiddennum);

b1 = gbest(inputnum*hiddennum+1:inputnum*hiddennum+hiddennum);

w2 = gbest(inputnum*hiddennum+hiddennum+1:inputnum*hiddennum

+hiddennum+hiddennum*outputnum);

b2 = gbest(inputnum*hiddennum+hiddennum+hiddennum*outputnum

+1:inputnum*hiddennum+hiddennum+hiddennum*outputnum+outputnum);

net.iw{1,1} = reshape(w1,hiddennum,inputnum);

net.lw{2,1} = reshape(w2,outputnum,hiddennum);

net.b{1} = reshape(b1,hiddennum,1);

net.b{2} = b2;

net.trainParam.epochs = 100;

net.trainParam.lr=0.1;

net.trainParam.goal=0.000001;

net = train(net,input_train,output_train);

1. **The PSO-BP codes for modelling the relationship among the provincial energy-related CO2 emissions and sum of DN values, which can be run by Matlab (R2017b).**

random_num = rand(1,630);

[value,index] = sort(random_num);

x_train = input(index(1:400),:)';

y_train = output(index(1:400));

x_test = input(index(401:630),:)';

y_test = output(index(401:630));

%Set the number of BPNN nodes

inputnum = 52;

hiddennum = 5;

outputnum = 1;

%Set the related parameters for PSO

sizepop = 10;% Population size

k = 50;% number of iterations

c1 = 2; c2 = 2;% learning factor

w = 0.8;% inertia factor

%Data normalization

[input_train,inputps] = mapminmax(x_train);

[output_train,outputps] = mapminmax(y_train);

%Determination of the number of optimization parameters

length = inputnum*hiddennum+hiddennum+hiddennum*outputnum+outputnum;

param = rand(sizepop,length);

speed = rand(sizepop,length);

%Establishing BPNN

net = newff(input_train,output_train,hiddennum);

%Initialization (individual best position lbest | global best position gbest | individual fitness fitness | group best fitness fitnessbest)

for i=1:sizepop

fitness(i,:) = func(param(1,:),inputnum,hiddennum,outputnum,net,input_train,output_train);

lbest(i,:) = param(i,:);

end

[value,index] = min(fitness);

gbest = param(index,:);% group extreme position (parameter)

fitnessbest = value;% group extreme fitness (minimum MSE)

% Parameter optimization

for T=1:k

for i=1:sizepop

speed(i,:) = w*speed(i,:)+c1*rand*(lbest(i,:)-param(i,:))+c2*rand*(gbest-param(i,:)) ;

param(i,:) = param(i,:)+speed(i,:);

fit = func(param(i,:),inputnum,hiddennum,outputnum,net,input_train,output_train);

if fit<fitness(i,:)

fitness(i,:) = fit;

lbest(i,:) = param(i,:);

end

if fit<fitnessbest

gbest = param(i,:);

fitnessbest = fit;

end

end

MSE(T,:) = fitnessbest;

end

% Build a model and predict (gbest is the best parameter)

w1 = gbest(1:inputnum*hiddennum);

b1 = gbest(inputnum*hiddennum+1:inputnum*hiddennum+hiddennum);

w2 = gbest(inputnum*hiddennum+hiddennum+1:inputnum*hiddennum+hiddennum

+hiddennum*outputnum);

b2 = gbest(inputnum*hiddennum+hiddennum+hiddennum*outputnum

+1:inputnum*hiddennum+hiddennum+hiddennum*outputnum+outputnum);

net.iw{1,1} = reshape(w1,hiddennum,inputnum);

net.lw{2,1} = reshape(w2,outputnum,hiddennum);

net.b{1} = reshape(b1,hiddennum,1);

net.b{2} = b2;

net.trainParam.epochs = 100;

net.trainParam.lr=0.1;

net.trainParam.goal=0.00001;

net = train(net,input_train,output_train);
